# A New Method for Estimating the Number of Undiagnosed HIV Infected Based on HIV Testing History, with an Application to Men Who Have Sex with Men in Seattle/King County, WA

**DOI:** 10.1371/journal.pone.0129551

**Published:** 2015-07-21

**Authors:** Ian E. Fellows, Martina Morris, Jeanette K. Birnbaum, Julia C. Dombrowski, Susan Buskin, Amy Bennett, Matthew R. Golden

**Affiliations:** 1 Fellows Statistics, San Diego, CA, United States of America; 2 Department of Sociology, University of Washington, Seattle, WA, United States of America; 3 Department of Statistics, University of Washington, Seattle, WA, United States of America; 4 Center for AIDS Research, University of Washington, Seattle, WA, United States of America; 5 Division of Allergy & Infectious Diseases, University of Washington School of Medicine, Seattle, WA, United States of America; 6 Department of Epidemiology, University of Washington School of Public Health, Seattle, WA, United States of America; 7 HIV/AIDS Program, Public Health Seattle/King County, Seattle, WA, United States of America; Rollins School of Public Health, Emory University, UNITED STATES

## Abstract

We develop a new approach for estimating the undiagnosed fraction of HIV cases, the first step in the HIV Care Cascade. The goal is to address a critical blindspot in HIV prevention and treatment planning, with an approach that simplifies data requirements and can be implemented with open-source software. The primary data required is HIV testing history information on newly diagnosed cases. Two methods are presented and compared. The first is a general methodology based on simplified back-calculation that can be used to assess changes in the undiagnosed fraction over time. The second makes an assumption of constant incidence, allowing the estimate to be expressed as a simple closed formula calculation. We demonstrate the methods with an application to HIV diagnoses among men who have sex with men (MSM) from Seattle/King County. The estimates suggest that 6% of HIV-infected MSM in King County are undiagnosed, about one-third of the comparable national estimate. A sensitivity analysis on the key distributional assumption gives an upper bound of 11%. The undiagnosed fraction varies by race/ethnicity, with estimates of 4.9% among white, 8.6% of African American, and 9.3% of Hispanic HIV-infected MSM being undiagnosed.

## Introduction

HIV prevention is increasingly focused on identifying infected persons and ensuring that they initiate and continue treatment[1]. The success of these efforts can be tracked through the HIV care cascade or care continuum[2], and many public health authorities now seek to measure each step in that continuum as a routine epidemiologic monitoring activity [3].

The first step in the care continuum—the proportion of HIV infected persons who have not yet been diagnosed—is perhaps the most challenging to estimate. Directly estimating this number requires HIV testing a representative sample of persons at risk for infection and identifying the fraction of infected persons who are unaware of their status. In the U.S., Centers for Disease Control and Prevention (CDC) investigators have adopted this direct approach as part of the National HIV Behavioral Surveillance system (NHBS) and their experience highlights some of the difficulties involved. The NHBS of men who have sex with men (MSM) relies on venue-based sampling, and the representativeness of the surveyed population is unknown. The system measures awareness of HIV status based on self-report, which is vulnerable to under-reporting by respondents who may be reluctant to reveal their HIV positive status to interviewers[4,5]. In addition, this approach is costly, labor intensive and not replicable by many local health jurisdictions.

Alternatively, estimates of the undiagnosed fraction can be indirectly obtained by using some form of back-calculation to estimate underlying HIV incidence. Variants of this method are increasingly being used for this purpose [6]. CDC has published back-calculation estimates for the undiagnosed fraction of MSM in the US of 19–26% (the range reflects differences by race/ethnicity) [7]. These estimates are based on an “extended back calculation” methodology and relies on surveillance data on the number of AIDS and HIV diagnoses in each year. The methodology requires a set of reasonably complex assumptions regarding testing frequency and the distributions of time from infection to AIDS diagnosis (see the web appendix to [8]), though the complete mathematical details of the model have not been published. As a result, local health departments currently do not have an accepted method for estimating the undiagnosed fraction of HIV infections in their area.

We present in this paper two new methods for estimating the undiagnosed fraction that are designed for use by local health departments. The first is a relatively simple back-calculation based approach which requires no assumptions about trends in HIV incidence, and the second is an even simpler formula that can be used when HIV incidence counts are stable. Both methods use data on HIV testing history for newly diagnosed cases, which already is, or can be, collected as part of routine public health activities. The calculations do not rely on AIDS diagnoses, so do not require assumptions regarding the distribution of time from infection to AIDS, which is a major potential point of model fragility for previous back-calculation approaches. The testing frequency distribution is estimated from patient reported testing history data, and thus avoids the need to assume a constant testing rate. Calculations are done using a package written in the programming language R, and both the R software and this package are open source and publicly available. We demonstrate the methods by estimating the undiagnosed fraction of HIV cases among MSM in King County, WA, overall and broken down by race.

## Materials and Methods

The method developed here relies on the date of a last negative test for all newly diagnosed HIV positive cases to estimate the distribution of time from infection to HIV diagnosis (TID), and then uses the TID to estimate the undiagnosed fraction. Since the methods were developed specifically to utilize the type of data that are often available in health departments, we start by describing the sources of HIV testing data available in our application setting in King County, WA. Approximately 85% of all cases of HIV in King County occur in MSM, so for this application we restrict our analysis to MSM.

### Sources of HIV testing data: King County, WA

HIV testing data were all collected as part of routine public health activities undertaken by Public Health—Seattle & King County (Public Health). Public Health has three sources of HIV testing history data: The enhanced HIV/AIDS reporting system (eHARS), the CDC treatment and testing history questionnaire (HIS), and data collected through HIV partner services (PS). The eHARS data only include dates of HIV negative tests that have verified documentation ascertained through review of medical records. HIS and partner services testing history data are collected by Public Health Disease Intervention Specialists (DIS) using standardized instruments, and the date of last test may be based on self-report by persons with newly diagnosed HIV infection and/or medical record reviews. Partner services data were only available for the period 2010–2012 and are based on client self-report. eHARS data has the advantage of including only verified information, but in some instances persons with HIV report having recent HIV negative tests that cannot be verified, and eHARS then records older but verifiable HIV test results. As part of our evaluation of these different sources of data, we used Pearson’s correlations to assess the agreement between the three data sources for time since last HIV negative test.

All data were anonymized and de-identified prior to analysis. As such, the IRB at the University of Washington does not define this as human subjects research.

### Methods and Assumptions

#### Estimating the time of last negative HIV test

This is directly estimated from the testing history data. We used the eHARS date when available, because it was a chart validated (if somewhat conservative) measure. In the absence of eHARS data, we used the most recent date observed in the HIS or partner services. If the day and/or month of the last HIV negative test was missing, we assumed that testing occurred in the middle of the month or on July 1, respectively.

#### Estimating the possible infection interval

For each individual, the period during which they were possibly infected was defined as the time between subjects’ last negative HIV test and their HIV diagnosis, with a maximum assigned value of 18 years. This maximum value is consistent with prior analyses suggesting that 95% of persons will develop AIDS within 18 years of infection [9]. When testing history data are missing, we defined the period of possible infection as their age at time of HIV diagnosis minus 16, the median age of sexual debut in the U.S.; here again we assume that no one is HIV infected for more than 18 years prior to diagnosis.

#### Estimating the distribution of time from infection to diagnosis (TID)

The methods start by estimating the population distribution of time from infection to diagnosis. This requires an assumption at the individual level about when infection occurs during the possible infection interval, so we conducted a sensitivity analysis using two different assumptions:

- Base Case Estimate: This assumes that infection occurred at a random point distributed uniformly during the period of possible infection. If some men test for HIV in response to specific risks, their TID will shift toward the end of the possible infection interval (as was observed in [10]), so this estimate is likely to be somewhat conservative.
- Upper bound Estimate: This assumes that HIV acquisition occurred at the beginning of the possible infection interval, i.e., the day after the last negative test. This represents the most conservative possible estimate of the time from infection to diagnosis consistent with the data.

In both cases we assume that the distribution of time from infection to diagnosis does not change appreciably from year to year, though testing frequency for individuals may vary. We tested this assumption using a Welsh ANOVA.

Using notation from survival analysis, let *F(t) = P(TID ≤ t)*, the cumulative hazard of diagnosis over time, and *S(t) = 1-F(t) = P(TID > t)*, the survival function for the probability of remaining undiagnosed over time. Suppose that we have observed *n* diagnoses with the length of the possible infection interval for the *i*th individual denoted by *x*_*i*_ We start by evaluating *F(t)* under each assumption. Under the upper bound assumption the estimate is simply the empirical CDF of the interval lengths

$${\hat{F}}_{UB}(t)=\frac{1}{n}\sum_{i=1}^{n}I(x_{i}\leq t)$$

where I is the indicator function, which is 1 if *x*_*i*_ ≤ *t* is true, and 0 otherwise. Under the base case assumption, the estimate is

$${\hat{F}}_{BC}(t)=\frac{1}{n}\sum_{i=1}^{n}max(1,\frac{t}{x_{i}})$$

From these cumulative hazard function estimates, we can calculate the discretized TID density, *f*_*t*_ = *F*(*t*+1)-*F*(*t*), the probability that an individual is diagnosed t quarters after infection, and *S*(*t*), under each assumption.

#### Direct calculation of undiagnosed cases assuming constant incidence

If both HIV incidence (*λ*) and the TID distribution are constant over time, a simple formula for estimating the number of undiagnosed can be used:

$$E(U)=\lambda \int_{0}^{\infty }S(t)\;dt=\lambda E(TID)$$ (1)

Under these conditions the expected number of new diagnoses will be equal to the incidence, so (Eq 1) can be estimated by substituting the average number diagnosed in each quarter for *λ*, and either the base case or upper bound estimate for *E(TID)*. A simple case that demonstrates the logic behind this calculation is provided in S1 Example.

#### Back calculation allowing for time-varying incidence

If incidence is not constrained to be constant, then back calculation must be used to estimate quarterly HIV incidence. Incidence counts are modeled as a (possibly time-varying) Poisson process with rate *λ*_*i*_ per quarter, using the standard convolution equation [11]:

$$E(D_{t})=\sum_{i\leq t}\lambda_{i}f_{t-i}$$

where *D*_*t*_ is the number diagnosed with HIV during quarter *t* and *f*_*t − i*_ is the discretized density function of the TID as defined above. The unknown incidence rates *λ*_*i*_ are traditionally estimated using an expectation-maximization (EM) algorithm [8]. Once the quarterly incidence rates have been estimated, the number undiagnosed at quarter *t* (*U*_*t*_) may be estimated as

$$E(U_{t})=\sum_{i\leq t}\lambda_{i}\left(\frac{1}{2}f_{t-i}+\sum_{k<t-i}f_{k}\right)$$

Each term in the outer sum represents the expected number infected during quarter *i* who are diagnosed after quarter *t* plus half the expected number of cases diagnosed during quarter *t*.

We made two refinements to the usual EM algorithm for fitting this model. First, since our data do not extend back to the beginning of the HIV epidemic but only back to 2006, we adjusted for our limited surveillance window. Second, we added a quadratic smoothing penalty to the likelihood to stabilize the parameter estimates and enforce the *a priori* assumption that any changes in incidence would be relatively gradual. The details of the fitting algorithm are described in S1 Details.

In contrast to the back calculation methods reviewed in [6], this method does not rely on AIDS diagnoses or on other biomarkers, and it does not require assumptions about the rates of testing, since the relevant testing interval is directly estimated from data. This considerably simplifies the back-calculation itself, and reduces the impact of assumptions related to disease severity, treatment and progression on the estimates.

#### Subpopulation estimates

Some sub-populations, such as groups defined by race or ethnicity, may have different testing behaviors and risks for infection. We tested for sub-population differences in time from last negative HIV test to diagnosis using Welsh ANOVAs, and estimated the number of undiagnosed persons by applying the methods outlined above to the subset of the data containing only those in the sub-population.

#### Estimates of the undiagnosed fraction of cases

The above equations provide an estimate of the number of undiagnosed cases of HIV infection. The proportion of cases with undiagnosed HIV infection is the estimated number of persons with undiagnosed HIV divided by the sum of the number persons with diagnosed and undiagnosed HIV infection. We used Public Health HIV surveillance data to estimate the number of persons with diagnosed HIV infection living in the area. Medical providers in Washington State are legally required to report all new cases of HIV infection to Public Health—Seattle & King County, and laboratories are required to report all positive HIV test results, all CD4 lymphocyte counts and all HIV RNA test results. Public health surveillance staff investigate all newly identified cases, and surveillance data integrate both in- and out-migration of persons to the area [12].

## Results

From 2006 through the end of 2012, 1522 MSM were diagnosed with HIV in King County, WA. Multiple sources of testing history data were often available, as shown in Table 1. eHARS, HIS, and HIV partner services data on date of last HIV negative test were available on 382 (25%), 1080 (71%), and 476 (31%) men, respectively. Combining data from the three sources, information on date of last HIV negative test was available for 1233 men (81%), including 101 (6.6%) men who were diagnosed with HIV at their first test; no data were available for the remaining 289 men (19%). When multiple sources of data were available, the HIS showed excellent agreement with the eHARS and partner services measures (correlations of 0.76 and 0.85 respectively) but the agreement between eHARS and partner services measures was less strong (correlation: 0.37), largely due to a cluster of outliers showing much more recent eHARS test date than the date reported in partner services interview. Given the hierarchical assignment, the last negative date was taken from eHARS for 382 men (34% of those with an observed last test date), from HIS for 670 men (59%, 131 of these had the same data in partner services), and from partner services for 80 men (7%).

**Table 1:** Overlap in HIV testing data sources from King County WA, and source used for analysis.

	Case also has:†			Used for analysis
Case has:	eHARS	HIS	PS	
Enhanced HIV/AIDS reporting system (eHARS)	**382**	346	113	382
Testing history questionnaire (HIS)		**1080**	456	670
Partner Services data (PS)††			**476**	80
Total with previous negative test				1132
Never Tested				101
Total with testing history information				1233
No testing history information				289
Total diagnosed				1522

Public Health Seattle/King County has three sources of testing history information. For cases that have more than one source of data, we use the most reliable source in this analysis (see text for more detail).

^†^ Diagonal elements represent the total number of newly diagnosed cases who had this source of testing information available.

^††^ Partner services data collection began in 2010, and were available for 69% of the 659 MSM newly diagnosed with HIV after this time. 165 cases had identical PS and HIS dates, and we count these as HIS in the “Used for Analysis” column.

**Fig 1:**
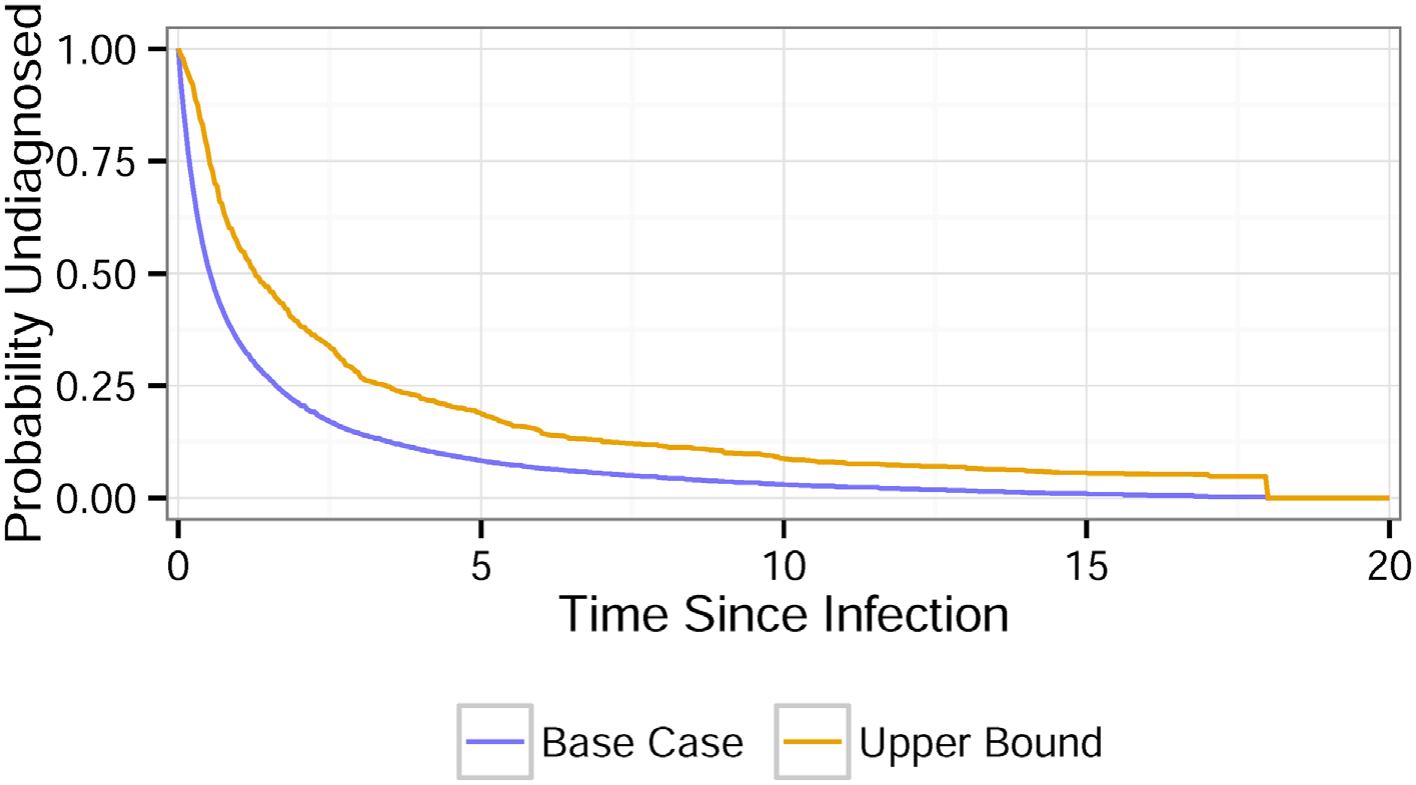
Distribution of time between infection and diagnosis (TID).

The estimated mean and median period of possible infection were 3.12 years and 1.25 years, respectively, with no statistically significant difference across years (F = 1.64 (6, 523), p > 0.1). Fig 1 shows the estimated TID distributions for the base case (Median = 0.5 years) and upper bound (Median = 1.3 years).

Two estimates of the population TID: the base case, which assumes a uniform distribution of the probability of infection in the possible infection interval, and the upper bound, which assumes infection occurs at the start of the possible infection interval, i.e., the day after the last negative test.

**Fig 2:**
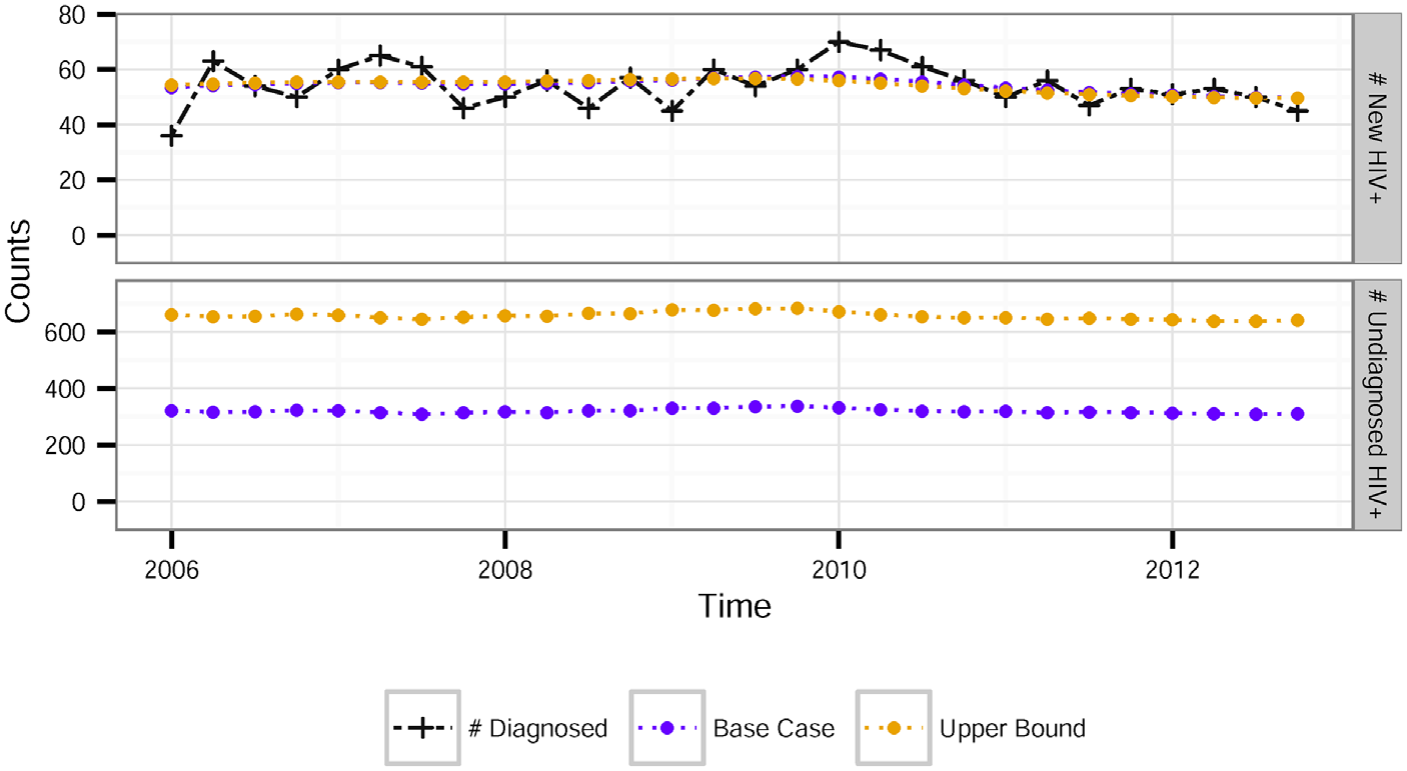
Observed and estimated quarterly HIV incidence among MSM in King County by quarter. (A) HIV incidence estimates and the observed number of diagnosed cases. The estimation uses a quadratic smoothing parameter of 0.1. (B) Estimates of the total number of undiagnosed HIV+ cases by quarter.

The back-calculation estimates of HIV incidence yielded almost identical quarterly counts for both the base case and upper bound TID models: 49.7–57.5 and 49.6–56.8 respectively. Fig 2A shows the estimated incidence counts over time for each model, along with the observed quarterly diagnosis counts. Both models find a relatively stable incidence over the 2006 to 2012 period, suggesting that the simpler constant incidence model can be used to estimate the undiagnosed fraction for these data.

Fig 2B shows the estimated number of undiagnosed cases of HIV among MSM based on each model. The number remains stable throughout the period of observation, ranging from 333–368 in the base case, and about double that, 662–713 for the upper bound. This base case estimates correspond to about 6% of HIV-infected MSM being undiagnosed; the upper bound to about 11%. Table 2 compares the back calculation based estimates (which do not assume constant incidence) to the estimates we can calculate directly by applying Eq 1 (assuming constant incidence).

**Table 2:** Estimates of the number and fraction of undiagnosed HIV cases among MSM in King County.

Distribution Assumption	Incidence Assumption	Number of MSM with HIV infection†	Number of MSM with undiagnosed HIV infection	Fraction Undiagnosed
Base case	None	5850–5884	333.5–367.8	5.7%-6.3%
	Constant	5863	346.7	5.9%
Upper bound	None	6178–6229	662.2–713.3	10.7%-11.4%
	Constant	6203.2	687.2	11.1%

^†^ Population size estimated as the sum of HIV-infected MSM thought to reside in King County, WA based on HIV surveillance data (n = 5516) plus the estimated number of undiagnosed cases.

**Fig 3:**
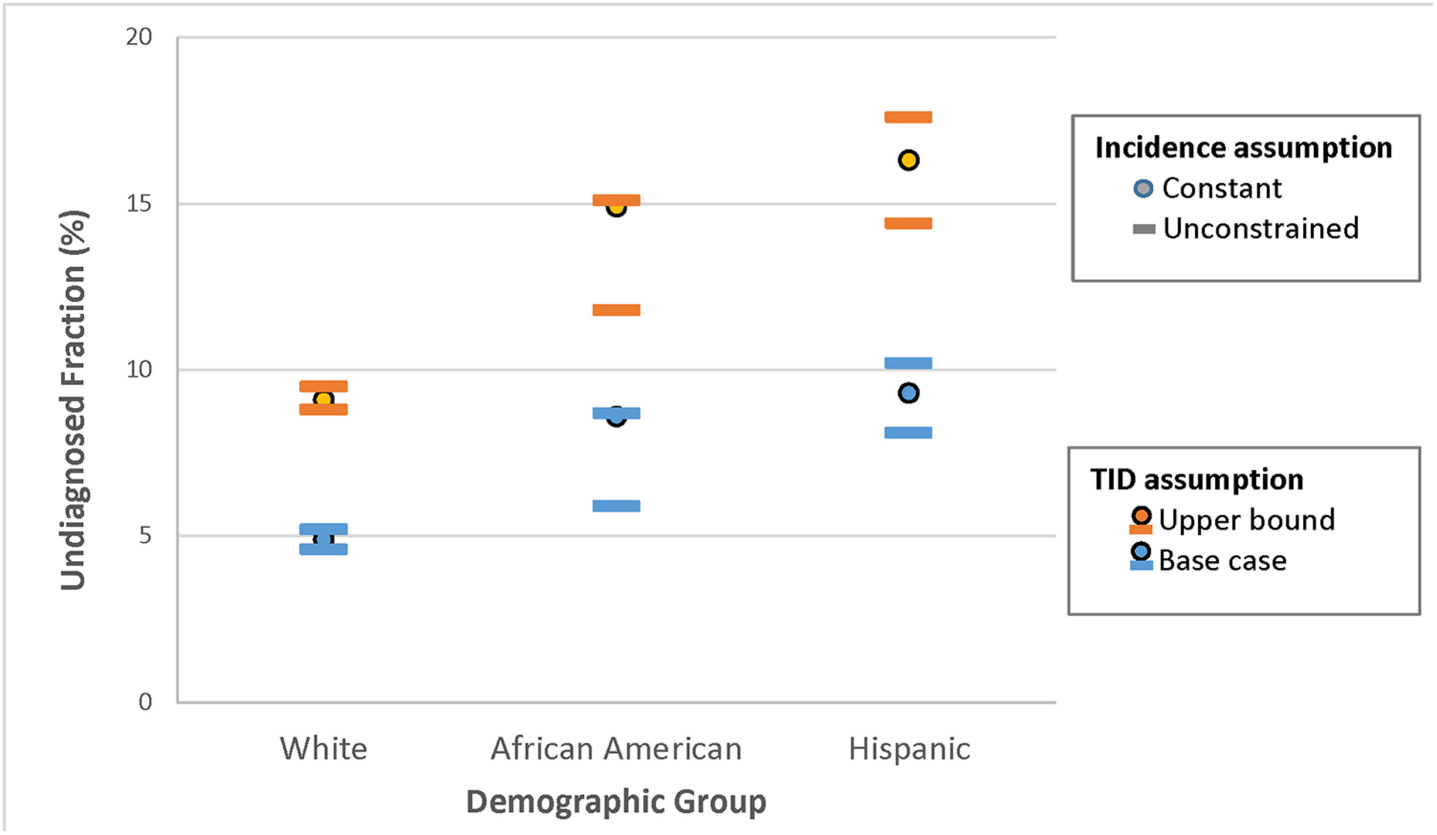
Racial/Ethnic disparities in the undiagnosed fraction with HIV. The plot shows the group-specific estimates under different assumptions: constant or time-varying incidence, and base case or upper bound estimate of the TID from Fig 1. The time-varying incidence estimates are summarized by the lowest and highest observed values from 2006–2012.

The testing history data show significant variation by race/ethnicity in the estimated time from infection to diagnosis. The mean possible infection interval estimates were 2.8, 4.3 and 3.4 years, for Whites, African Americans and Hispanics respectively (F = 4.5 (2,199), p < 0.05). The medians are 1.2, 1.8 and 1.5 respectively. As shown in Fig 3, this contributes to relatively large differences in estimates of the undiagnosed fraction between White MSM, and MSM of color.

Using the base case model and assuming constant incidence, we estimate that 4.9% of White HIV-infected MSM, 8.6% of African American HIV-infected MSM, and 9.3% of Hispanic HIV-infected MSM in King County are undiagnosed (the full table of estimates for each model broken down by race/ethnicity is provided in S1 Table). It may seem counterintuitive that the undiagnosed fraction would be slightly higher among Hispanics than African Americans when their mean TID is lower. The reason is that shape of the TID for Hispanic MSM is distinctively different: their early diagnoses are later than both Whites and African Americans, while their later diagnoses are more frequent than whites, but less extreme than African Americans. For example, 11% of African Americans had periods greater than 15 years, versus 6% among Hispanics, but 25% of African Americans had tested negative within 6 months of diagnosis, versus 20% for Hispanics.

## Discussion

We present a new approach to estimating the proportion of persons with undiagnosed HIV infection based on HIV testing history data. Applying the methods to data from King County, WA, our best estimate suggests that about 6% of HIV-infected MSM are undiagnosed. If we use the most conservative assumptions that are consistent with the observed data, the upper bound overall estimate is 11%. Under both models, the estimated undiagnosed fraction is almost twice as high among African Americans and Hispanics than among Whites.

Our estimates for King County, WA are much lower than the most recent national estimates: using an extended back calculation method, CDC investigators estimated that in 2008 19% of HIV-infected MSM were undiagnosed [7]. The extent to which our findings reflect true differences between King County and the U.S. as a whole, versus differences due to methodology is not clear. Ideally, we would compare our results to estimates generated using CDC’s extended back calculation method [8,12] [7]. However, at present, the details of that method are not available in sufficient detail to undertake such a comparison. However, several other findings suggest that at least some part of the difference may be real. First, based on laboratory reported surveillance data, Public Health currently estimates that 68% of HIV diagnosed persons in the area are virologically suppressed compared to national estimate of 31% [3,12]. Second, among MSM participants in the 2008 cycle of NHBS, 15% of HIV-infected MSM in Seattle vs. 44% of MSM nationally were reported to be unaware of their HIV status [13]. And finally, in the 2014 cycle of NHBS, only 7.5% of 81 HIV-infected MSM did not report having a prior HIV diagnosis (Thiede H, personal communication, national 2014 NHBS data are not currently available.) While the validity of NHBS findings on this subject have been questioned [4] and the NHBS sample may not be representative of all HIV-infected MSM, we believe that the very large difference observed within 2008 NHBS suggests that there is a real difference of some magnitude between King County, WA and the U.S. as a whole, while the 2014 NHBS data are consistent with our finding that very few HIV-infected King County MSM are undiagnosed.

Our findings have several important limitations. First, our estimate is based on HIV testing history data with varying levels of possible measurement error. Just over 30% of the cases draw the last negative test date from the eHARS system, and eHARS records the last test date that can be validated against medical records. This places an upper bound on the last negative HIV test date, but it may not be the most recent test, so this may bias the TID toward conservative estimates. For the remainder of the cases (66%), TID is based on a self-reported date of last test. The validity of these data are not known. When more than one data source was present, we found good agreement between eHARS and the HIS self-report measures (the HIS measures are used for most of the remaining cases), but less agreement with the PS measures (used for the final 7%). Errors in self-reports could result in either an over estimate or an underestimate of the undiagnosed fraction. Second, our base case model assumes that individuals are infected uniformly between last negative test and first positive. If some people test in response to a recent risk exposure [10], our base case assumption is also conservative, and the undiagnosed fraction would be lower than what we report here. It would be possible to extend this method to incorporate such data, if the surveillance system collected such information. Finally, over 80% of the cases in our data had some information on the date of last negative test; the estimates from this method are likely to be more uncertain, and less useful, in populations for which testing prior to diagnosis is uncommon, or data on HIV testing history are unavailable. In the vignette that accompanies the open source code released with this paper, we simulate the impact of lower prior test rates, and find the increase in uncertainty to be modest even when half of the cases lack a test prior to diagnosis.

These methods could be useful to public health agencies interested in monitoring their local HIV care continuum. We developed them in response to a request from our Center for AIDS Research Northwest Regional Public Health Consortium, a group convened to advance the integration of public health practice and university research in our region. The approach is designed to be relatively simple to implement and understand, and to integrate local data to create locally relevant estimates. Compared to the range of back calculation methods currently used for estimating the undiagnosed fraction [6], our approach depends on fewer, and more straightforward assumptions, and requires simpler data and statistical methods. It does not use data on AIDS diagnoses, so it does not incur the well-known uncertainties associated with assumptions about the distribution of time from infection to AIDS. Instead, it relies on testing history data. The King County experience demonstrates that it is feasible to routinely collect testing history data as part of HIV surveillance and partner services, and our method can be used with as little as one year’s worth of data (though the trend analysis would clearly require data over time). For health departments that do not currently collect such data, this may provide an impetus for adding questions related to HIV testing history to partner services and surveillance investigations.

We have not presented confidence intervals (which would be conditional on the statistical model), choosing to focus instead on the model uncertainty. The model uncertainty arising from the key assumption—the distribution of time from infection to diagnosis in the test window interval—was explored with sensitivity analysis to identify a true upper bound that was consistent with the observed data. Bootstrap confidence intervals could be developed, but we expect that in most contexts, the diagnosed cases in a surveillance system are close to a census of all diagnosed cases, so the uncertainty associated with the model assumptions will be substantially larger than the uncertainty due to sampling.

The statistical methods presented here are implemented using a package written in R, an open source software environment. The package, and a simulated data set that matches the key characteristics of the data used in this paper will be available on GitHub when this paper is published. We have also developed a simple graphical user interface for the package based on Shiny [14]; it too is open source, and allows end users to access the package functionality through point and click options via a browser-window; no familiarity with R programming is required.

## Supporting Information

S1 Table HIV Incidence and undiagnosed fraction estimates broken down by race/ethnicity. Estimates of the number of undiagnosed HIV cases among MSM in King County stratified by ethnicity. * Sum of cases thought to reside in King County based on HIV surveillance data (N = 4188, 458, and 572 respectively) and the estimated number of undiagnosed cases.
(TEX)

S1 Example Constant Incidence Calculation. This example uses a very simple case to show the logic behind the constant incidence calculation.
(TEX)

S1 Details Additional details on the backcalculation algorithm. This provides more detail on the basic backcalculation algorithm, accounting for limited surveillance windows, and the quadratic smoothing penalty.
(TEX)
