# Correction: A New Method for Estimating the Number of Undiagnosed HIV Infected Based on HIV Testing History, with an Application to Men Who Have Sex with Men in Seattle/King County, WA

**DOI:** 10.1371/journal.pone.0135878

**Published:** 2015-08-12

**Authors:** Ian E. Fellows, Martina Morris, Jeanette K. Birnbaum, Julia C. Dombrowski, Susan Buskin, Amy Bennett, Matthew R. Golden

Supporting Information files S1 Table, S1 Example, and S1 Details are incorrectly published in raw TeX format rather than PDF format. Please see the formatted PDF files here.

## Supporting Information

S1 Table HIV Incidence and undiagnosed fraction estimates broken down by race/ethnicity. Estimates of the number of undiagnosed HIV cases among MSM in King County stratified by ethnicity. * Sum of cases thought to reside in King County based on HIV surveillance data (N = 4188, 458, and 572 respectively) and the estimated number of undiagnosed cases.
(PDF)

S1 Example Constant Incidence Calculation. This example uses a very simple case to show the logic behind the constant incidence calculation.
(PDF)

S1 Details Additional details on the backcalculation algorithm. This provides more detail on the basic backcalculation algorithm, accounting for limited surveillance windows, and the quadratic smoothing penalty.
(PDF)
