# Correction: A New Method for Estimating the Number of Undiagnosed HIV Infected Based on HIV Testing History, with an Application to Men Who Have Sex with Men in Seattle/King County, WA

**DOI:** 10.1371/journal.pone.0197707

**Published:** 2018-05-16

**Authors:** Ian E. Fellows, Martina Morris, Jeanette K. Birnbaum, Julia C. Dombrowski, Susan Buskin, Amy Bennett, Matthew R. Golden

In the Methods section, the second equation under the heading titled “Estimating the distribution of time from infection to diagnosis (TID)” should say “min” instead of “max.” Please view the complete, corrected equation here:

$${\hat{F}}_{BC}(t)=\frac{1}{n}\sum_{i=1}^{n}min(1,\frac{t}{x_{i}})$$

## References

[1] Fellows IE, Morris M, Birnbaum JK, Dombrowski JC, Buskin S, Bennett A, et al (2015) A New Method for Estimating the Number of Undiagnosed HIV Infected Based on HIV Testing History, with an Application to Men Who Have Sex with Men in Seattle/King County, WA. PLoS ONE 10(7): e0129551 https://doi.org/10.1371/journal.pone.0129551 26196132 10.1371/journal.pone.0129551 PMC4510124

