# Supplementary material for: Correction: A New Method for Estimating the Number of Undiagnosed HIV Infected Based on HIV Testing History, with an Application to Men Who Have Sex with Men in Seattle/King County, WA
Source: PLoS One. 2015 Aug 12;10(8):e0135878. doi: 10.1371/journal.pone.0135878 (PMC4534198; doi:10.1371/journal.pone.0135878)
Supplement: S1 Table — Estimates of the number of undiagnosed HIV cases among MSM in King County stratified by ethnicity. * Sum of cases thought to reside in King County based on HIV surveillance data (N = 4188, 458, and 572 respectively) and the estimated number of undiagnosed cases. (PDF) [file pone.0135878.s001.pdf]

S1. HIV Incidence and undiagnosed fraction estimates broken down by race/ethnicity

Ethnicity	TID Scenario	Incidence Model	Incidence Count (per quarter)	MSM Undiagnosed	Total HIV infected MSM*	Percentage Undiagnosed
White (n=1035)	Upper bound	Varying	33.1-40.4	402-441	4590-4629.2	8.8%-9.5%
		Constant	37	420	4608	9.10%
	Base case	Varying	33.3-40.5	203-229	4391-4417	4.6%-5.2%
		Constant	37	214	4402	4.90%
African American (n=129)	Upper bound	Varying	3.4-7.2	61-81	519-539	11.8%-15.1%
		Constant	4.6	80	539	14.90%
	Base case	Varying	3.4-7.2	29-44	487-502	5.9%-8.7%
		Constant	4.6	423	501	8.60%
Hispanic (n=230)	Upper bound	Varying	3.6-10.2	96-122	668-694	14.4%-17.6%
		Constant	8.2	112	684	16.30%
	Base case	Varying	4.4-10.3	51-65	623-637	8.1%-10.2%
		Constant	8.2	58	631	9.30%

Table 1. Estimates of the number of undiagnosed HIV cases among MSM in King County stratified by ethnicity. (* Sum of cases thought to reside in King County based on HIV surveillance data (N= 4188, 458, and 572 respectively) and the estimated number of undiagnosed cases)
