# Supplementary material for: Correction: A New Method for Estimating the Number of Undiagnosed HIV Infected Based on HIV Testing History, with an Application to Men Who Have Sex with Men in Seattle/King County, WA
Source: PLoS One. 2015 Aug 12;10(8):e0135878. doi: 10.1371/journal.pone.0135878 (PMC4534198; doi:10.1371/journal.pone.0135878)
Supplement: S1 Example — This example uses a very simple case to show the logic behind the constant incidence calculation. (PDF) [file pone.0135878.s002.pdf]

S2. A Constant Incidence Example Calculation

It may be useful for some readers to see a simple illustrative example calculation using equation 1. Suppose that we observe 4 subjects, with time since last negative tests of $\{.25, .75, .25, 1\}$ years respectively and we have an incidence rate of 4 cases per year. Then if we use the upper bound estimate of the TID distribution then the probability that an infected individual remains undiagnosed for more that t is

$$S(t) = P(TID > t) = \begin{cases} 1, & \text{if } t < .25 \\ .5, & \text{if } .25 \leq t < .75 \\ .25, & \text{if } .75 \leq t < 1 \\ 0 & \text{if } t \geq 1 \end{cases},$$

and our estimated number of undiagnosed can then be calculated as

$$E(U) = E(Y) \int_0^\infty S(t)dt = \lambda E(TID) = 4 * (1 * .25 + .5 * .5 + .25 * .25) = 2.25.$$
