# Supplementary material for: Correction: A New Method for Estimating the Number of Undiagnosed HIV Infected Based on HIV Testing History, with an Application to Men Who Have Sex with Men in Seattle/King County, WA
Source: PLoS One. 2015 Aug 12;10(8):e0135878. doi: 10.1371/journal.pone.0135878 (PMC4534198; doi:10.1371/journal.pone.0135878)
Supplement: S1 Details — This provides more detail on the basic backcalculation algorithm, accounting for limited surveillance windows, and the quadratic smoothing penalty. (PDF) [file pone.0135878.s003.pdf]

S3. Additional details on the backcalculation algorithm

1 Back-Calculation Background

Let Y_i be the number of individuals diagnosed with HIV at time $i \in \{1, ..., T\}$, and X_i be the (unobserved) number of infected at time i. It is assumed that the X_i are independently distributed Poisson with expectation λ_i, and thus the Y_i are also independently distributed Poisson with means $\sum_{j=0}^{i} \lambda_i f_{j,i-j}$, where $f_{j,d}$ is the probability that an individual infected at time j is diagnosed at time $j + d$.

Given the $f_{j,d}$ distribution, following the methodology of [**?**], which was adapted to the HIV/AIDS setting from work done in image cleaning for PET scans (See [**?**] and references therein), we can express the log likelihood as

$$\ell(\lambda|Y = y) = \sum_i y_i log(\sum_{j=0}^{i} \lambda_i f_{j,i-j}) - \sum_{j=0}^{i} \lambda_i f_{j,i-j}.$$

Given the high dimensional nature of λ, maximizing this likelihood directly is impractical. Instead, we define a latent variable $N_{i,j}$ to be the number of infected at time i who are diagnosed at time j, which is distributed Poisson with mean $\lambda_i f_{j-i}$. The joint likelihood is then written as

$$\ell(\lambda|Y = y, N = n) = \sum_{i=1}^{T} \sum_{d=0}^{T-i} n_{i,i+d} log(\lambda_i f_d) - \lambda_i f_d$$

This likelihood can then be maximized via the EM algorithm, the E-step of which is

$$E(\ell(\lambda|Y = y, N = n)|Y = y, \lambda = \lambda') = \sum_{i=1}^{T} \sum_{d=0}^{T-i} \frac{\lambda'_i f_d}{\sum_{r=0}^{i+d} \lambda'_r f_{i+d-r}} log(\lambda_i f_d) - \lambda_i f_d,$$

which yields a fairly straightforward update in the M-step

$$\lambda_k^{(i+1)} = \frac{\lambda_k^{(i)}}{\sum_{d<T-k} f_d} \sum_{d+k<T-k} \frac{y_{k+d} f_d}{\sum_{r<k+d} \lambda_r^{(i)} f_{k+d-r}}. \quad (1)$$

1.1 Estimating the number of undiagnosed

Given a fit model, we may estimate the number of undiagnosed individuals at the mid-point of time interval j (U_j) as

$$E(U_j) = \sum_{i<j} \lambda_i (\frac{1}{2} f_{i,j-i} + \sum_{k>j-i} f_{i,k})$$

where $\sum_{k>j-i} f_{i,k}$ is the expected number of individuals infected at time i diagnosed after time j and $f_{i,j-i}$ is the expected number of individuals infected at time i and diagnosed during time period j.

2 Accounting for limited surveillance windows

If the historical data Y goes back to the beginning of the HIV epidemic, such as in [**?**], then Equation 1 is the correct update to use. However, if the diagnosis data is only

available after a certain time t_0 after the start of the epidemic, then some of the Y_i are actually missing. This changes the E-step to

$$\begin{aligned} Q(\lambda|Y=y,\lambda') &= E(\ell(\lambda|Y=y,N=n)|Y_{t_0}=y_{t_0},...,Y_T=y_t,,\lambda=\lambda') \\ &= \sum_{i=0}^{T}\sum_{d+i<t_0}\lambda'_i f_d log(\lambda_i f_d)+\sum_{d=t_0}^{T-i}\frac{\lambda'_i f_d}{\sum_{r=0}^{i+d}\lambda'_r f_{k+d-r}}log(\lambda_i f_d)-\sum_{d=0}^{T-i}\lambda_i f_d \end{aligned}$$

and the M-step update equations become

$$\lambda_k^{(i+1)}=\lambda_k^{(i)}(a_k+\frac{c_k}{b_k})$$

where $a_k=\frac{\sum_{d-k<t_0}f_d}{b_k}$, $b_k=\sum_{d<T-k}f_d$ and $c_k=\sum_{d=t_0-k}^{T}\frac{y_{k+d}f_d}{\sum_{r<k+d}\lambda_r^{(i)}f_{k+d-r}}$.

3 Smoothing via quadratic penalties

It is well known that the maximum likelihood estimate yields noisy solutions [?], whereas we expect a priori that the mean infection rates, year to year display smooth trends. [?] proposed incorporating a smoothing step in the EM algorithm. This method was applied to HIV/AIDS data by [?]. More modern work from the PET literature focused on adding a penalty (or equivalently a prior distribution) to the log likelihood enforcing smoothness (see [?] and references therein). The likelihood with a quadratic smoothing penalty is defined as

$$\ell_p(\lambda|Y=y,N=n)=\ell(\lambda|Y=y,N=n)-\gamma\sum_{i=2}^{T}(\lambda_i-\lambda_{i-1})^2$$

where γ is a positive smoothing parameter. The M-step of the EM algorithm then becomes

$$Q_p(\lambda|Y=y,\lambda')=Q(\lambda|Y=y,\lambda')-\gamma\sum_{i=2}^{T}(\lambda_i-\lambda_{i-1})^2.$$

If $\gamma>0$, this penalty represents an a priori belief that the epidemic is in a stable state with constant incidence, which is constant with the current state of the epidemic in the United States. [?], for example, report stable infection rates from the early 1990s through 2007. [?] have also applied smoothing penalties to HIV/AIDS data in order to remove noise, however they utilized a penalty that was quadratic in the log scale and implied an a priori belief that the trend in HIV infection rate is either growing or declining at an exponential rate.

3.1 Update algorithm using numeric root finding

Having the quadratic term couples the λ parameters, making the simple update equations inapplicable. However, it is possible to find a solution by maximizing the likelihood numerically. The gradient of the penalized likelihood is

$$\begin{aligned} \frac{\delta Q_p}{\delta\lambda_k} &= \frac{1}{\lambda_k}\lambda'_k(a_k b_k+c_k)-b_k-2\gamma(\lambda_k-\lambda_{k-1})+2\gamma(\lambda_{k+1}-\lambda_k) \\ &= \frac{1}{\lambda_k}\lambda'_k(a_k b_k+c_k)-b_k-4\gamma\lambda_k-2\gamma(\lambda_{k+1}-\lambda_{k-1}) \end{aligned}$$

and its hessian is a banded matrix with

$$\begin{aligned}
\frac{\delta^2 Q_p}{\delta^2 \lambda_k} &= \frac{1}{\lambda_k^2}\lambda'_k(a_k b_k + c_k) - 4\gamma \\
\frac{\delta^2 Q_p}{\delta\lambda_k \delta\lambda_{k+1}} &= -2\gamma \\
\frac{\delta^2 Q_p}{\delta\lambda_k \delta\lambda_{k-1}} &= 2\gamma.
\end{aligned}$$

Each step of the EM algorithm is therefore defined as the lambda such that the root of $\frac{\delta Q_p}{\delta \lambda_k}$ is attained. This can be computed via the Newton-Raphson algorithm using the banded hessian matrix. An efficient implementation of this is present in the R package rootSolve [**?**].

References
